# Anesthesia and cancer recurrence: an overview

**DOI:** 10.1186/s44158-022-00060-9

**Published:** 2022-07-20

**Authors:** Etrusca Brogi, Francesco Forfori

**Affiliations:** Department of Anesthesia and Intensive Care, University of Pisa, Via Paradisa 2, 56124 Pisa, Italy

**Keywords:** Anesthesia, Stress factors, Cancer, Cancer recurrence, Outcome

## Abstract

Several perioperative factors are responsible for the dysregulation or suppression of the immune system with a possible impact on cancer cell growth and the development of new metastasis. These factors have the potential to directly suppress the immune system and activate hypothalamic-pituitary-adrenal axis and the sympathetic nervous system with a consequent further immunosuppressive effect.

Anesthetics and analgesics used during the perioperative period may modulate the innate and adaptive immune system, inflammatory system, and angiogenesis, with a possible impact on cancer recurrence and long-term outcome. Even if the current data are controversial and contrasting, it is crucial to increase awareness about this topic among healthcare professionals for a future better and conscious choice of anesthetic techniques.

In this article, we aimed to provide an overview regarding the relationship between anesthesia and cancer recurrence. We reviewed the effects of surgery, perioperative factors, and anesthetic agents on tumor cell survival and tumor recurrence.

## Introduction

Surgery represents one of the leading treatments for the therapeutic management of several kinds of tumors. However, at the same time, surgery can have a direct and an indirect effect on tumor cell survival leading to tumor recurrence. Surgery can lead to the release of cancer cells into the bloodstream during tumor manipulation with consequent metastatic spread to distant organs [1]. Furthermore, even with clear resected surgical margins, minimal residual disease may remain and flourish with consequent local or lymphatic spread [2]. Additionally, several perioperative factors, such as inflammatory response to surgery, hypothermia, blood transfusion, tissue hypoxia, hyperglycemia, post-operative pain, can create a state of relative immunosuppression [3, 4]. Stress factors also have the potential of activating the systemic inflammatory response and enhancing tumor growth, with consequential increasing the risk of metastatic recurrence [5]. Then, the aforementioned factors have also the potential of creating an appropriate microenvironment for tumor growth through the release of hormonal mediators (i.e., catecholamines, prostaglandins), cytokines (e.g., interleukin-6, IL-4 and IL-10, TGF-β) and the upregulated expression of the transcription factor hypoxia-inducible factor 1-alpha (i.e., HIF1A) with consequent enhancement of angiogenesis pathways, cell proliferation, and the metastatic ability of cancer cells [6–8]. Not only, surgical stress can also trigger the hypothalamic-pituitary-adrenal axis and the sympathetic nervous system which in turn also regulates the immune response with the consequent further suppression of cell immunity [9].

Likewise, anesthesia techniques may affect metastatic progression of tumor cells [10]. In fact, anesthetic drugs can play a modulatory effect on the immune system, on systemic inflammatory response, on neuroendocrine stress response and on cancer signaling pathways [11–13]. The influence of the anesthetic technique on neuroendocrine, inflammatory, and immune responses during surgery can alter local and systemic immunity with consequent boosting the tumor growth factors production and loco-regional recurrence and metastasis [14]. Even more, anesthetic-analgesic drugs seemed also to mediate the expression of specific genes or molecular pathways involved in the control of differentiation, cell growth, and of tumor progression [11]. Interestingly, evidence suggested that propofol may have a potential antitumor effect due to the regulation of mRNA expression [15]. Several preclinical and clinical studies have already shown the potential impact of anesthetics and adjuvants on cancer recurrence and survival [10]. What seems to emerge from the existing literature is that opioids can suppress the humoral immune response and can have pro-angiogenic effects, whereas regional anesthesia techniques have been associated with lower rates of cancer recurrence [16–18]. Even more, it seemed that total intravenous anesthesia (TIVA) was associated with improved recurrence-free survival in comparison to volatile anesthesia [19]. Thus, evidence is arising about the possible relation between anesthesia technique and cancer recurrence, however, a huge limitation to the current literature is represented by the impossibility of evaluating the effect of each single drug on cancer recurrence, since anesthesia requires a combination of different classes of anesthetics (i.e., hypnotic, analgesic). Consequently, further studies are needed on this topic.

Accordingly, it is crucial for healthcare personnel to consider the possible relation and implication between anesthesia, perioperative stress factors and cancer for a future better and conscious choice of anesthetic technique with the goal of improving cancer outcome. In this article, we aimed to provide an overview regarding the relationship between anesthesia and cancer recurrence. We reviewed the effects of surgery, perioperative factor, and anesthetic agents on tumor cell survival and tumor recurrence.

### Perioperative metastasis

Perioperative stress factors trigger physiological responses that in turn can create an appropriate microenvironment for the growth of pre-existing micro-metastatic, for the formation of new ones and for their spread [20]. Several perioperative variables (i.e., the inflammatory response to surgery, hypothermia, and blood transfusion) represent important risk factors responsible for creating a state of relative immunosuppression and of increasing vulnerability to cancer recurrence.

Perioperative metastasis survival and growth are mediated through various mechanisms [21]:

- Increase shedding of cancer cells due to mechanical manipulations of the tumor during surgery [1];
- Activation of inflammatory response [22];
- Modulation of immune function [23];
- Triggering the neuroendocrine and paracrine stress responses [24];
- Activation of pro-angiogenic signaling pathways [25];
- Expression of specific genes and/or molecular pathways [26].

Metastasis can occur through transcoelomic, lymphatic, and/or hematogenous routes. Transcoelomic spread refers to the diffusion of cancer cells to the peritoneal cavity, due to the migration of a primary cancer of the abdomen/pelvis or due to the systemic spread of another kind of primary cancer [27]. During abdominal and pelvic operation, surgical manipulation can be responsible for intraperitoneal seeding [28]. Even more, lymphatic network is commonly increased in solid tumors, especially in tumor margin and peritumor area and lymph flow that drains tumors is often increased, with increased interstitial fluid pressure and consequent altered lymphatic drainage [29–31]. Consequently, mechanical disruption and manipulation of the cancer during surgery may facilitate the dissemination of tumor cells also through lymphatic routes [32]. In fact, surgical incision may be responsible for endothelial disruption and consequent increase in the hydrostatic and oncotic pressures, thus favoring migration of cancer cells in the lymphatic network and subsequent dissemination. Additionally, physiological response to surgical stress led to an overexpression of lymphangiogenic factors (i.e., vascular endothelial growth factor (VEGF), prostaglandins, and platelet-derived growth factor (PDGF)) with consequent further enhance of tumor dissemination [33–35]. Surgery may also increase the hematic release of circulating tumor cells (CTC); the levels of CTC were found to be increased during different kind of surgeries [36–39]. Not all the CTC are able to seed with the consequent formation of distant metastasis. To accomplish this process, CTC have to escape circulating immune defenses and to migrate and invade fertile zone to colonize. Several inflammatory mediators and hypoxic conditions are responsible of creating vulnerable areas where CTC can migrate and proliferate: the so-called pre-metastatic niche [40].

The activation of inflammatory system due to surgical stress lead to the migration of macrophages, neutrophils, fibroblasts and mesenchymal stem cells on the site of the surgery [41]. These cells secrete several factors (e.g., VEGF, PDGF, epidermal growth factor-EGF, prostaglandin, matrix metalloproteinases (MMP)), responsible for promoting cancer growth, lymphangiogenesis, angiogenesis, and consequent dissemination [42]. Prostaglandins play an important role in increasing the metastatic invasiveness of cancer cells through the activation of several receptors (e.g., B2-adrenergic, and cyclooxygenase-2 receptors) [43, 44]. Even more, MMP and VEGF are responsible for favoring tumor cell adhesion, angiogenesis, and invasiveness of cancer cells [42, 45]. Interestingly, platelet seemed to play an important role in immune escaping of cancer cells [46]. In fact, micro-clot formation can protect CTC from natural killer (NK), from cell-mediated detection, and promotes CTC adhesion to the endothelium. Even more, activated platelets can release soluble mediators (i.e., transforming-growth factor beta -TGF-β, PDGF and adenosine triphosphate) with important effects on immune system: modulation of the NK activity and of the vascular permeability [47]. Furthermore, local and systemic immune responses to surgery lead to pro-inflammatory and immunosuppressive consequences with deeply suppression of cell-mediated immunity (CMI) [6]. The consequent immunosuppression is due to the release of several mediators such as cytokines (e.g., Interleukin-6), with an inhibitory effect on NK activity. Remarkably, several trials have found an increased level of Th2 lymphocytes and decrease level of Th1 lymphocytes with altered Th1/Th2 ratio during cancer surgery [48]. These responses may represent another important aspect to consider regarding the relation between perioperative stress response and immunosuppression.

The activation of neural signaling is induced not only by surgical tissue trauma but also by other stress factors (e.g., hypothermia, tissue hypoxia, and patient anxiety). The activation of neural signaling (i.e., the sympathetic nervous system and the hypothalamic–pituitary–adrenal axis) led to the release of stress hormones (i.e., catecholamines, opioids, and glucocorticoids) with important consequences on cancer cell invasiveness [49]. The consequent hormonal storm stimulates inflammatory and immunologic response. Afferent nerves from the site of tissue damage triggers the activation of the HPA axis and sympathetic nervous system with consequent secretion of ACTH, cortisol, catecholamines, aldosterone, vasopressin, and glucagon. Cortisol are natural steroid hormones that bind the transcription factor glucocorticoid receptor (GR). The hypersecretion of cortisol lead to the upregulation of anti-inflammatory protein and downregulation of pro-inflammatory protein expression. Even more, cortisol influences the adaptive and innate immunity systems. Because of increased cortisol production, the number of circulating monocytes, macrophage and dendritic cells are reduced. Even more, another important consequence is represented by reduction of circulating T cells, with a shift from a pro-inflammatory Th1 phenotype to an anti-inflammatory Th2 phenotype. Glucocorticoids also effects the expression of genes that regulate the inflammatory response (i.e., NF-KB and AP-1) and inhibits the activation, proliferation, and production of immunoglobulins by B cell lymphocyte [50]. Even more, the activation of the neuroendocrine response is also responsible of changing tumor microenvironment, and remodeling lymphatic and blood vasculature [51]. All these processes are implied in tumor recurrence. Stress hormones were reported to downregulate NK, cytotoxic T lymphocytes activity, and macrophage motility/phagocytosis [52, 53]. Furthermore, catecholamine bind β-adrenoceptors on cell surface with activation of calcium-cAMP signaling and consequent enhancement of pro-metastatic factors transcription (e.g., HIF, VEGF, and MMP) [54]. Beta-adrenoreceptors have been found in several cancer cells (i.e., breast, prostate, lung, liver) [54]. The activation of these signaling pathways leads to increase tumor cell growth and their invasiveness.

**Fig. 1:**
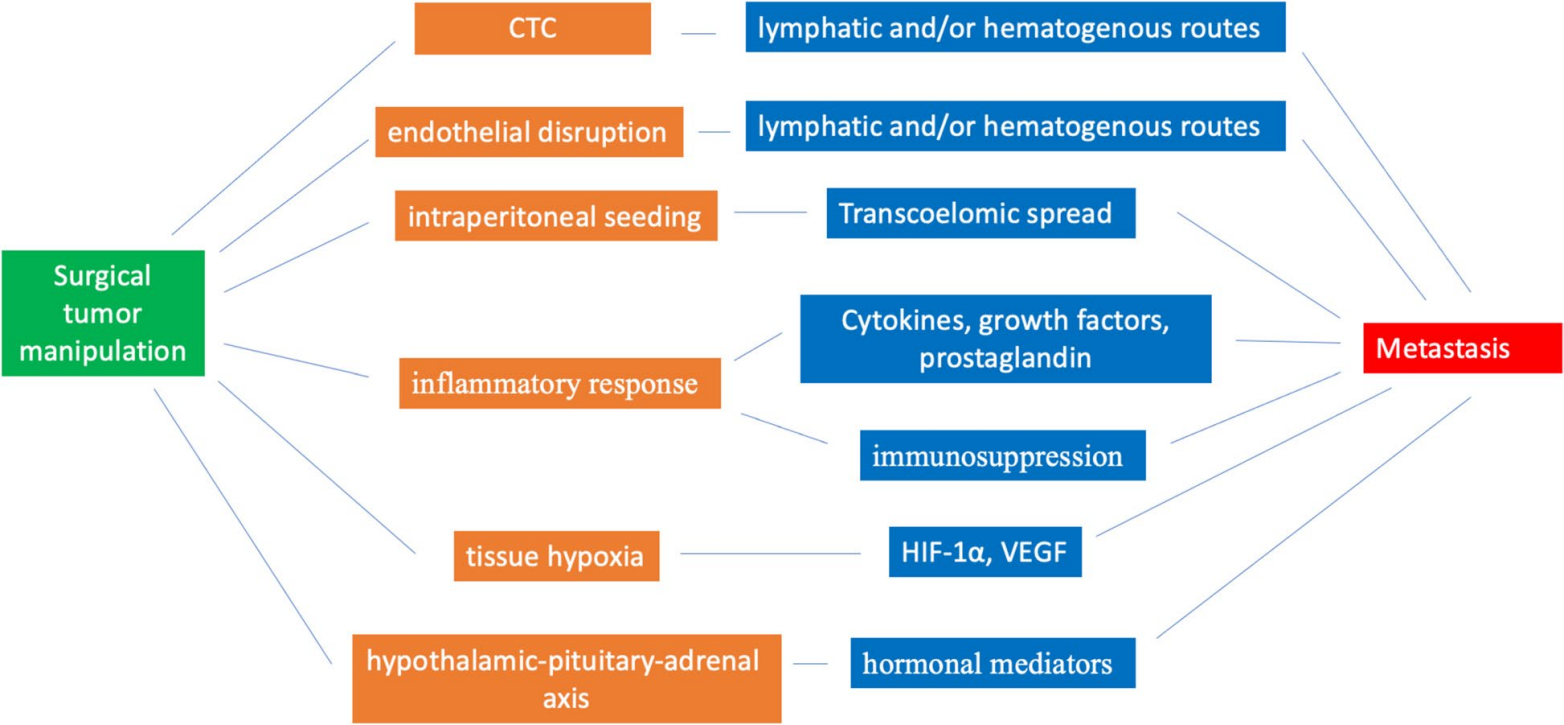
Schematic representation of perioperative metastasis due to surgical manipulation

Finally, another important aspect is represented by the possible correlation between stress response and expression of specific genes or molecular pathways with the consequent changes in the cell signaling [26, 55, 56]. The epigenetic modification of gene expression involved during surgery is due to DNA methylation, histone modifications, chromatin, and noncoding RNAs (ncRNAs) remodeling [57]. Furthermore, the disruption of local vasculature during surgery, lead to hypoperfusion, ischemia, and hypoxia. Hypoxia stimulates the upregulated expression of the transcription factor hypoxia-inducible factor 1-alpha (i.e., HIF1A) with consequent promotion of angiogenesis, cell proliferation, and metastasis [58]. Furthermore, HIF promotes the secretion of angiogenic factors (e.g., VEGF and angiopoietin 2) with a further effect on tumor progression and metastatic spread [59]. The level of HIF1A has been correlated with tumor progression, metastatic spread and outcome [60]. Hypoxic conditions lead also to increased production of reactive oxygen species (ROS). The consequent oxidative stress can trigger several transcription factors (i.e., NF-κB, AP-1, p53, HIF-1α, PPAR-γ, β-catenin/Wnt, and Nrf2) that in turn lead to the expression of growth factors, inflammatory cytokines and chemokines [61]. The effect of surgery and of anesthetic techniques on cancer recurrence are summarized in Tables 1 and 2. A schematic representation of perioperative metastasis due to surgical manipulation is presented in Fig. 1.

**Table 1:** Effects of surgery on cancer recurrence

*Effects of surgery on cancer recurrence*		
	Action	Consequences
*Direct effect* on tumor cell survival	Surgical tumor manipulation	Release of cancer cells into the bloodstream ➔ metastatic spread to distant organs
	Surgical tumor manipulation	Intraperitoneal seeding➔ Transcoelomic spread
	Surgical tumor manipulation and incision	Endothelial disruption ➔ increase hydrostatic and oncotic pressure➔dissemination of tumor cells through lymphatic routes
	Minimal residual disease in surgical margins	Local or lymphatic spread
	Action	Consequences
*Indirect effect* on tumor cell survival	Physiological response to perioperative stress factors	Activating the systemic inflammatory response➔ migration of macrophages, neutrophils, fibroblasts on the site of the surgery ➔ Release of cytokines, growth factors and prostaglandin➔ promoting cancer growth, lymphangiogenesis, angiogenesis, and consequent dissemination
	Physiological response to perioperative stress factors	Activating the systemic inflammatory response➔ state of relative immunosuppression➔ immune escaping of cancer cells➔appropriate microenvironment for tumor growth
	Physiological response to perioperative stress factors	Trigger the hypothalamic-pituitary-adrenal axis and the sympathetic nervous system➔ release of hormonal mediators➔ enhance tumor growth
	Physiological response to perioperative stress factors	Expression of specific genes and/or molecular pathways➔ promotion of angiogenesis, cell proliferation, and metastasis
	Physiological response to perioperative stress factors	Activation of pro-angiogenic signaling pathways➔ increasing the metastatic invasiveness

**Table 2:** Effects of anesthetics on cancer recurrence

*Effects of anesthetics on cancer recurrence*	
*Type of anesthetics*	*Effects*
*Volatile anesthetics*	-Pro-inflammatory and immunosuppressive action -Reduces Th1/Th2 ratio -Impairs NK cell activity -Induces T cell and B cell apoptosis -Upregulation of hypoxia-inducible factors (HIF-1α, HIF-2α,) -Increase transcription of pro-metastatic factors (VEGF, angiopoietin-1, proteases MMP-2, and MMP-9) -Enhanced tumor cell proliferation -Increase angiogenesis, and cell migration
*Intravenous anesthetics*	-Anti-inflammatory and immunosuppression properties -Suppression of prostaglandin and inflammatory cytokine production -Inhibition of cyclooxygenase (COX) activity -Stimulate the proliferation of NK cells -Increase expression of granzyme B and IFNγ -Increase cytotoxic T lymphocyte activity -Does not affect the Th1/Th2 ratio -Modulate genetic signaling pathways -Inhibits histone acetylation
*Ketamine, Thiopental*	-Suppress the activity of NK cells -Induce apoptosis in lymphocytes -Inhibits the functional maturation of dendritic cells -Reduce the synthesis of pro-inflammatory cytokines
*Opioids*	-Modulate wound healing -Immunosuppression effects -Inhibits natural killer cell activity -Inhibits responses of T and B cells to mitogens -Inhibits antibody production -Promotes lymphocyte apoptosis, -Reduces the differentiation of T cells -Inhibits phagocytic activity -Inhibits of the release of cytokine/ chemokine production
*Local anesthetics*	-Activates apoptotic pathway -Inhibits tumor cell growth and migration -Increases the activity of NK -Increases the number of T-helper (Th) cells -Preserves Th1/Th2 cells ratio -Preserves IFN-gamma concentrations -Modulates gene expression -Increases IL-4 levels -Decreases IL-10, IL-8, TNF-alfa production
*NSAIDs and COX-2 inhibitors*	-Inhibits the cyclooxygenase 1 and the cyclooxygenase 2 -Reduces prostaglandin synthesis
*Paracetamol*	-Inhibits prostaglandin endoperoxide H2 synthase and cyclooxygenase activity

### Anesthetic agents

#### Volatile and intravenous anesthetics

The increasing interest in the impact of anesthetics and cancer progression has stimulated several in vivo and in vitro studies on the relation between different kinds of anesthetics used during surgery and cancer development and progression [48, 62]. Even if the evidence is conflicting, halogenated anesthetics seemed to present several pro-inflammatory and immunosuppressive effects that can have an important impact on enhancing metastasis formation [63]. Volatile anesthetic agents are implied in the upregulation of hypoxia-inducible factors [64]. Several trials are showed that the exposure of cancer cells to isoflurane and sevoflurane led to upregulation of HIF-1α, HIF-2α, growth factor and increase transcription of pro-metastatic factors (VEGF, angiopoietin-1, proteases MMP-2 and MMP-9, insulin-like growth factor IGF-1) which enhanced tumor cell proliferation, increased angiogenesis, and cell migration [65, 66]. Furthermore, halogenated anesthetics inhibit the activity of the immune system; reduces Th1/Th2 ratio, impairs NK cell activity, induces T cell and B cell apoptosis [67–69]. Consequently, the volatile anesthetic may promote immunosuppression and the creation of a pro-malignant environment that supports the growth of residual cancer cells.

On the other hand, propofol presents anti-inflammatory and immunosuppression properties [70–72]. Several studies have shown that propofol could inhibit adhesion, migration, invasiveness of cancer cells and induce apoptosis [73, 74]. Propofol presents anti-inflammatory properties through the suppression of prostaglandin and inflammatory cytokine production and the inhibition of cyclooxygenase (COX) activity [75]. Even more, propofol may prevent immunosuppression through the preservation of NK cell function. Not only propofol preserved NK activity, it seemed that propofol could also stimulate the proliferation of NK cells through the increased expression of granzyme B, IFN-γ, and activating surface receptors (e.g., CD16, NKp30, NKp44, and NKG2D) [76–78]. In fact, increased NK cell infiltration of tumors is reported after the administration of propofol. Furthermore, propofol could increase cytotoxic T lymphocyte activity and does not affect the Th1/Th2 ratio [79].

Propofol may also modulate genetic signaling pathways with important consequences on carcinogenesis:

- Inhibition of HIF-1α protein synthesis induced by hypoxia [80];
- Inhibition of the mRNA expression of MMP-2 and MMP-9 and p38 MAPK signaling (signaling pathway regulating proliferation, cell motility, and survival) [81];
- Inhibition of the NF-κB pathway [82];
- Downregulation of S100A4 in endothelial cells and suppression of VEGF expression from cancer cells with consequent anti-angiogenic effects [83, 84];
- Upregulating miRNA expression (tumor suppressors and by inhibiting the expression of miRNAs that works as oncogenes) [85];
- Inhibiting histone acetylation [86].

Noteworthy, signaling pathways are not usually independent and participate in a crosstalk to create a regulatory network. Consequently, propofol may affect several pathways with important regulation on genes expression. Propofol with its anti-inflammatory and pro-immunity effects has been suggested to have a positive impact on long-term survival and cancer outcome [87–90]. However, no unified conclusion has been reached and further evidence is needed to come to a clear conclusion. In 2019, a randomized controlled trial was published comparing the incidence of metastatic breast cancer recurrence in patients who received regional anesthesia and propofol versus general anesthesia with volatile anesthetic sevoflurane and opioid analgesia [91]. The studies included 2108 women who underwent breast surgery. Cancer recurrence was similar between the groups. Contrarily, a 2019 meta-analysis by Yap et al. analyzed the effects of anesthetics on cancer recurrence and survival [19]. The study included ten trials. The authors found that TIVA was associated with improved recurrence-free survival.

In 2021, Ramirez et al. performed a review describing how drugs may regulate important function on immune and cancer cells [92]. The authors presented several preclinical and clinical studies and explained the effects of anesthetics on cancer cells. The authors presented 21 retrospective and 4 RCTs studies comparing the effects of TIVA versus volatile anesthesia. They also presented 28 retrospective and 9 RCTs studies assessing the effects of regional anesthesia on long-term outcome. Preclinical evidence showed that volatile anesthesia regulates important function in cancer cells and that they can directly modify intracellular signal involved in proliferation, migration and invasion. The authors concluded that “…whether volatile anesthetics have a deleterious effect on cancer recurrence and survival remains a controversial issue…”; however, Ramirez explained how “…volatile anesthesia regulate important function in cancer cells.”. This evidence suggested that anesthetics may play a potential impact on cancer recurrence, at least from a cellular point of view. Of course, we cannot speculate that the result of preclinical studies could be translated into clinical practices.

Finally, ketamine and thiopental present immune effects. Thiopental inhibits the function of neutrophils and NK [93]. Ketamine may suppress the activity of NK cells, induce apoptosis in lymphocytes and inhibits the functional maturation of dendritic cells [94]. Ketamine may also reduce the synthesis of pro-inflammatory cytokines, (e.g., IL-6, TNF-α) [95]. However, the evidence regarding the relation between ketamine and thiopental and cancer is scarce and far to be conclusive.

#### Opioids

Increasing evidence suggests that, beyond their primary analgesic function, opioids present several physiological effects. Opioids modulate wound healing and cancer progression through their endothelial action and through their influence on angiogenesis [17]. Furthermore, opioids are known to act on the immune system with immunosuppression effects [16, 96]. Through the mu-opioid receptor (MOR) or non-opioid receptors (toll-like receptors) expressed by immune cells, opioids play their direct effect on the immune system, inhibiting natural killer cell activity, inhibiting responses of T and B cells to mitogens and antibody production [97–100]. Furthermore, opioids can inhibit several neutrophils and macrophages activity: inhibition of phagocytic activity and inhibition of the release of cytokine/chemokine production [101]. Moreover, opioids act indirect effects on the immune system through the sympathetic nervous system and the hypothalamic-pituitary-adrenal axis [102, 103].

The interplay between opioids and cancer, however, is complex and far to be understood deeply. It was also observed that neutrophils, macrophages and T cells also release endogenous opioid peptides with consequent reduction of inflammation and pain through the binding of peripheral opioid receptors [96, 104]. Noteworthy, it is important to take into account that the control of pain may have a beneficial indirect effect on immunity. The balance between the immunosuppressive effect of the opioid and the reduction of immunosuppression of pain is difficult to foresee [105].

In brief, different kinds of opioids seemed to act different effects in in vitro/in vivo model:

- Morphine: suppresses the activity of NK cells, promotes lymphocyte apoptosis, reduces the differentiation of T cells, and stimulates angiogenesis [99];
- Fentanyl: decrease the activity of NK cells and increase the number of regulatory T cells [106];
- Sufentanil: decrease the activity of NK cells, increase the number of regulatory T cells, inhibits leukocyte migration [107];
- Alfentanil: decreases the activity of NK cells [108];
- Remifentanil: suppress the activity of NK cells and lymphocytic proliferation [109].

Interestingly, methyl-naltrexone, an opioid antagonist, seemed to inhibit tumor cell invasion and implantation, while continuous infusion of MNTX decreases primary tumor growth and development of lung metastasis [110].

#### Local anesthetics

The implementation of regional anesthesia/analgesia techniques seemed to have a positive impact on reducing cancer recurrence via several mechanisms [111]:

- Reduces the stress response to surgery (via pain control or sympathetic block) and reduces the levels of cortisol, β-endorphin, and epinephrine [112, 113];
- Reduces the need for opioids or volatile agents (indirect effect);
- Activates apoptotic pathway [114];
- Inhibits tumor cell growth and migration [115];
- Increases the activity of NK [116];
- Increases the number of T-helper (Th) cells, preserved the ratio of Th1 to Th2 cells [117];
- Preserves IFN-gamma concentrations [118];
- Modulates gene expression, DNA demethylation [119];
- Increases IL-4 and decreasing IL-10, IL-8, TNF-alfa [120].

Besides the possible beneficial mechanism triggered by regional anesthesia, there is no strong evidence regarding the effect of regional anesthesia on cancer recurrence. Xu et al. evaluated the effects of epidural anesthesia-analgesia on recurrence-free survival after lung cancer surgery*.* The authors compared two groups: general anesthesia versus general anesthesia and regional anesthesia groups [121]. The authors concluded that regional anesthesia did not improve recurrence-free survival compared with general anesthesia alone. In both groups, general anesthesia was induced with propofol, sufentanil, and rocuronium while anesthesia was maintained with propofol and/or sevoflurane (with or without nitrous oxide inhalation). Even more, dexmedetomidine was given at the discretion of anesthesiologists. Consequently, due to the high heterogeneity of drugs administered (propofol, sevoflurane, opioids, dexmedetomidine), it was not possible to come to any conclusion regarding general anesthesia. It was impossible of evaluating the effect of each single drug on cancer recurrence. Similarly, in Du et al., the authors concluded that regional anesthesia did not improve recurrence-free survival compared with general anesthesia alone [122]. Even more, general anesthesia was induced with midazolam, propofol, sufentanil, and rocuronium and maintained with either intravenous, inhalation, or combined. A 2015 meta-analysis including 10 studies showed improved overall survival when neuraxial analgesia was used in radical prostatectomy [123]. On the other hand, as aforementioned mentioned, in 2019 a randomized controlled trial did not find any difference in cancer recurrence between the groups receiving regional anesthesia and propofol versus general anesthesia with volatile anesthetic sevoflurane and opioid analgesia [91]. Several studies were conducted on this topic; however, due to the heterogeneity of the trials, it is difficult to draw any conclusion from the existing literature [118, 124–126].

#### NSAIDs, COX-2 inhibitors, paracetamol, alpha-2 adrenoceptor agonists

Other drugs commonly used in the perioperative period:

- *NSAIDs and COX-2 inhibitors:* represented the most widely painkiller used for the management of perioperative analgesia. NSAIDs inhibit the cyclooxygenase 1 (COX-1) and the cyclooxygenase 2 (COX-2) enzymes with consequent anti-inflammatory, analgesic and antipyretic effects. Several trials have already shown the potential benefits of NSAIDs in the prevention of human cancer [127]. Above all, the long-term use of daily low-dose aspirin has been already related to the risk reduction of several kind of cancers: from colon, breast, lung, and prostate cancer [127, 128]. COX is frequently overexpressed in several cancers with important effects on cancer progression with an important contribution in tumorigenesis [127, 129–131]: increased production of prostaglandins, inhibition of apoptosis and promotion of angiogenesis, increased cell motility and invasion and modulation of inflammation and immune function [132, 133]. NSAIDs inhibit cyclooxygenase enzymes, leading to reduction of prostaglandin synthesis (i.e., prostaglandin E2, PGE_2_) and promote immune responses [134]. In particular, PGE_2_ plays a crucial role in promoting cancer progression; enhancement of cellular proliferation, promotion of angiogenesis, inhibition of apoptosis, stimulation of invasion/motility, and suppression of immune response [44]. Nevertheless, NSAIDs can be administered in combination with opioids or with paracetamol to increase the analgesic efficacy and to reduce the daily consumption of opioids [135]. Consequently, the possible survival benefits of receiving NSAIDs may be also due to their opioid-sparing effects of the usage of multimodality therapy in the perioperative settings [136].
- *Paracetamol:* inhibits prostaglandin endoperoxide H2 synthase and cyclooxygenase activity with pain-relieving and antipyretic properties. However, paracetamol has no anti-inflammatory effects. Paracetamol can be administered in combination with opioids or NSAIDs to increases the analgesic efficacy and reduce daily morphine consumption [137]. Analyzing the current literature, the relationship between paracetamol usage and cancer recurrence are conflicting: increased risks for urinary tract cancers and decreased risk for ovarian cancer [138, 139]. However, the results reached so far have been inconsistent.
- *Alpha-2 adrenoceptor agonists:* dexmedetomidine and clonidine are alpha-2 adrenoceptor agonists mainly used for sedation and as part of multimodal opioid-sparing analgesia. Alfa-adrenoceptors are found to be expressed in breast cancer, both epithelial and stromal cells [140]. Consequently, alfa-modulators may affect cancer progression and recurrence. However, evidence is scarce regarding the relation between dexmedetomidine and/or clonidine and cancer recurrence and far to be conclusive [141–143].

## Discussion and conclusions

Overview articles represent a useful aid for addressing bias and concerns or to put light on the insufficiency of the current literature and to stimulate further research in a particular field. We decided to provide an overview only on the impact of anesthetic techniques and surgery on cancer recurrence because the current data are controversial and contrasting. Our aim was to summarize content from several articles and provide the reader with a general understanding of the possible relation between anesthetics and cancer.

It is also important to highlight that, up to now, the heterogeneity of the factors implied in cancer recurrence during surgery are high and the heterogeneity of the current literature on cancer and anesthesia would make impractical, or at least hard, to summarize and to come to any kind of conclusion. Not only the anesthetic technique but also several perioperative factors can influence immune surveillance and inflammatory responses and they may favor proliferation of metastasis. Furthermore, the impact of anesthetics technique depending on the type of cancer could make the discussion confusing considering the vast and divergent literature available on this topic. This would made even more difficult to come to any kind of conclusion.

Another important limitation is represented by the fact that it is impossible to evaluate the effect of each single drug on cancer recurrence, since anesthesia requires a combination of different classes of drugs (i.e., hypnotic, analgesic). The difference in baseline characteristics between groups (i.e., ASA), the different concentration of volatile anesthetics used in the clinical studies, the different duration of the surgery and the extension of surgical incision (minimally invasive vs. open surgery) represented important confounding factors. Even more, the majority of the data looking at the relationship of these techniques and cancer outcome in different kind of tumor originates from retrospective studies.

Surely, evidence is arising about the possible impact of anesthesia technique, perioperative period, cancer recurrence and long-term outcome. Even if the current data are controversial and contrasting, it is crucial to increase awareness about this topic among healthcare professionals for a future better and conscious choice of anesthetic techniques. Consequently, further trials are needed for a deeper understanding of the aforementioned mechanisms and on the actual impact of anesthetic techniques on the long-term survival. At this stage of the clinical research, we think that share awareness represents the major goal in an informative way.

## Data Availability

Not applicable.
